# Assessing the Impact of Medication and Stenting on the Quality of Life of Patients With Chronic Pancreatitis: A Prospective Study

**DOI:** 10.7759/cureus.50106

**Published:** 2023-12-07

**Authors:** Ravindrasingh Rajput, Durga Prasad S, C Ganesh Pai

**Affiliations:** 1 Physiology, American University of Antigua College of Medicine, St. John's, ATG; 2 Pharmacology, American University of Antigua College of Medicine, St. John's, ATG; 3 Gastroenterology, Kasturba Medical College, Manipal, IND

**Keywords:** analgesic potential, fatigue, role functioning, global health scores, pain management, european organization for research and treatment of cancer quality of life questionnaire (eortc qlq-c30), endoscopic stenting, pharmacotherapy, quality of life (qol), chronic pancreatitis

## Abstract

This research aimed to assess the effect of pharmacotherapy alone versus the combination of pharmacotherapy and endoscopic stenting on the quality of life (QoL) outcomes of chronic pancreatitis patients. Chronic pancreatitis, an inflammatory disease, often presents with persistent pain, affecting patients' quality of life. Thirty patients treated either with pharmacotherapy alone or with the addition of endoscopic stenting were analyzed. The European Organization for Research and Treatment of Cancer Quality of Life Questionnaire (EORTC QLQ-C30) was used to gather data on the patients' QoL. Results showed that both treatment groups experienced improvements in global health, role functioning, fatigue, and abdominal pain scores over follow-ups. Specifically, the stenting group saw notable enhancements in global health and role functioning. The study's conclusions provide valuable insights into the potential benefits of both treatments, with stenting offering significant improvements in certain QoL parameters. However, the sample size and source limit generalizability, suggesting the need for more extensive research across diverse settings.

## Introduction

In today's world, significant efforts are being made to discover a cure for chronic diseases such as chronic pancreatitis and various cancers; however, finding a definitive cure remains a challenge. Simultaneously, there is a growing focus on enhancing the quality of life (QoL) and providing maximum comfort to patients, aiming for a life free from symptoms. Chronic diseases typically begin with one or two symptoms but often lead to a multitude of direct and indirect manifestations [1]. These conditions impact not only health-related aspects but also social, familial, and financial dimensions [2]. Consequently, to address the frustration and suffering caused by the crisis on multiple fronts, a well-designed questionnaire study can identify treatment options that benefit patients from various perspectives. Such a study can guide healthcare organizations in assessing the cost-effectiveness of available therapeutic interventions and facilitate effective interventions to improve overall quality of life [3].

Chronic pancreatitis is an inflammatory disease characterized by progressive and irreversible destruction of pancreatic tissue. Its clinical course is marked by dynamic fibrosis that progressively affects the pancreas. Studies conducted in Europe, North America, and Japan have reported an incidence of chronic pancreatitis ranging from two to 10 cases per 100,000 population per year [4]. This disease is more prevalent among males, with an average age of onset of around 40 years [3].

Typically, chronic pancreatitis presents as persistent and unrelenting pain, often accompanied by episodic flares. Nausea and vomiting are common symptoms associated with the pain. Due to the discomfort, patients frequently avoid eating, which can result in significant weight loss, especially if steatorrhea is present [5]. As the disease progresses, pancreatic endocrine and exocrine dysfunction may develop, leading to various complications. Approximately one-third of patients develop mild overt diabetes mellitus [6]. Consequently, the natural history of chronic pancreatitis is generally associated with a challenging prognosis.

The primary focus of most therapies for chronic pancreatitis is to reduce exocrine pancreatic secretion and allow the pancreas to rest; however, this approach is generally ineffective. Treatment goals include alleviating pain, addressing pancreatic insufficiency, and managing complications.

Medical therapy primarily involves the use of pancreatic enzyme supplements and antioxidants [7,8]. Studies such as "A pilot study of the antioxidant effect of curcumin in tropical pancreatitis" by Durgaprasad et al. highlight the potential antioxidant effect of curcumin in the context of tropical pancreatitis, suggesting the role of natural compounds in managing the disease [9]. If pain persists despite these measures, analgesics may be prescribed, and in severe cases, opioid analgesics may be necessary. Pancreatic endotherapy, including procedures such as pancreatic stenting, has gained popularity for the treatment of chronic pancreatitis [10]. However, the safety and effectiveness of these interventions continue to be evaluated. After undergoing total pancreatectomy with islet autotransplantation (TPIAT), notable enhancements in the quality of life were observed [11].

To ensure effective intervention, it is essential to assess the nature, frequency, and severity of the patient's pain and its impact on daily activities. Encouraging patients to maintain a pain log and utilizing quality of life instruments, such as questionnaires, can aid in this assessment process [12].

The primary objective of this study was to evaluate the impact on quality of life outcomes in chronic pancreatitis patients treated with pharmacotherapy alone versus those who underwent endoscopic pancreatic stenting in addition to pharmacotherapy.

## Materials and methods

The study protocol received approval from the institutional review board of Kasturba Medical College (KMC), Manipal University (IAEC/KMC/06/2007-2008) and was conducted in accordance with the principles outlined in the Declaration of Helsinki. Written informed consent was obtained from all participating patients prior to their inclusion in the study. In this prospective study, patients were divided into two treatment groups. The first group received pharmacotherapy alone, consisting of pancreatic enzymes (lipase, amylase, and protease), proton pump inhibitors or H2 antihistamines, and analgesics. The second group underwent endoscopic pancreatic duct stenting, with concomitant pharmacotherapy.

Quality of life (QoL) data were collected using the European Organization for Research and Treatment of Cancer Quality of Life Questionnaire (EORTC QLQ-C30). Assessments were conducted at baseline, as well as at the third and sixth month follow-ups, for a subgroup of patients. The EORTC QLQ-C30 questionnaire, which has been validated and translated into three languages (English, Kannada, and Malayalam), was utilized in the study. The overall duration of the study was two years.

Inclusion criteria

The inclusion criteria for our study were carefully defined to ensure a focused and relevant participant group. Firstly, we limited the age range of participants to those between 18 and 65 years, allowing us to study adults across a broad age spectrum. Secondly, eligibility was contingent upon a confirmed diagnosis of chronic pancreatitis. This diagnosis needed to be established through a comprehensive clinical evaluation, corroborated by imaging findings and diagnostic endoscopy, ensuring that only patients with a clear and confirmed diagnosis were included. Finally, an essential criterion was the patients' willingness and commitment to attend all scheduled follow-up visits. This requirement was crucial for maintaining consistent and reliable longitudinal data, which is pivotal for assessing the progression of the disease and the efficacy of treatments over time.

Exclusion criteria

For our study, we established specific exclusion criteria to ensure the clarity and integrity of our findings. Patients diagnosed with pancreatic cancer were excluded to maintain a focus solely on chronic pancreatitis. Individuals presenting with ascites, a condition that could complicate the clinical picture, were also not included. It was important to isolate the pain symptoms attributable to chronic pancreatitis; hence, patients experiencing pain from other causes were excluded. Additionally, we required participants to have a clear understanding of the study language and no cognitive impairments, to ensure accurate communication and comprehension of the study procedures and requirements. Lastly, we excluded patients suffering from comorbid illnesses, with the exception of diabetes mellitus, as the presence of other significant medical conditions could interfere with the study's focus on chronic pancreatitis and its direct impacts.

Questionnaire selection

In this study, the EORTC QLQ C-30 questionnaire was chosen based on previous research indicating its validity and usefulness for evaluating the effectiveness of different treatment options in chronic pancreatitis [13]. The QLQ C-30 questionnaire comprises both multi-item scales and single-item measures. It is part of the EORTC quality of life questionnaire system, designed to assess the health-related quality of life (QoL) of cancer patients participating in international clinical trials [14].

For this study, both in-patients and out-patients who were proficient in either Kannada or Malayalam, the local languages, were selected. The questionnaire was provided in both languages, and the participants completed it in the presence of the investigator. In addition to the QLQ C-30 questionnaire, other factors such as age, sex, duration of illness, and history of alcohol intake were also considered.

The QLQ C-30 questionnaire [15] consists of multiple scales measuring different aspects. These include functional scales, symptom scales, a global health status/QoL scale, and single-item measures. Each scale contains a unique set of items, with no item appearing in multiple scales. Scores for all scales and single-item measures range from 0 to 100, where a higher score indicates a higher level of response. Therefore, a high score on a functional scale reflects better functioning or health, while a high score on a symptom scale or item indicates a higher level of symptomatology or problems.

Modes of administration

Various modes of administration, such as personal interviews and telephone interviews, can potentially introduce bias when conducting quality of life assessments [16]. A study comparing face-to-face interviews with telephone interviews reported mean directional difference scores of 2.5 points [17]. Given this information, we believe that it is reasonable to combine the results obtained from both personal and telephone interviews in a descriptive study, as it allows for a comprehensive assessment of quality of life. Assessments were conducted at the baseline with two follow-ups, i.e., one at the end of three months and another at the end of six months.

Statistical analysis

The baseline scores, as well as the scores from the two follow-up assessments conducted at three-month intervals, were presented using suitable charts and tables. A comparison of the baseline characteristics will be performed using an independent t-test. The progress of patients in each group over the course of six months was analyzed using the Friedman-Wilcoxon test, utilizing the Statistical Package for the Social Sciences (SPSS) version 23 software package (IBM SPSS Statistics, Armonk, NY).

## Results

A total of 34 patients diagnosed with chronic pancreatitis were initially enrolled in the study, and written informed consent was obtained from each participant. However, four patients were later excluded from the study due to their inability to attend the scheduled follow-up visits. Therefore, a total of 30 patients were included in the final analysis. Additionally, one patient unfortunately passed away after the first follow-up assessment, resulting in a missing data point that will be addressed according to the guidelines provided by the EORTC questionnaire.

Out of the 30 patients, 18 were in the pharmacotherapy group, while 12 were in the stenting group. Among the participants, 86.7% (26) were male, and 13.3% (4) were female. The overall mean age of the patients was 37.47±13.40 years. The mean age for the pharmacotherapy group was 36.27±14.58 years, and for the stenting group, it was 39.25±11.76 years. However, there was no statistically significant difference between the two groups in terms of age (p=0.561).

The overall median duration of illness among the patients was two years. For the pharmacotherapy group, the median duration of illness was 1.75 years, while for the stenting group, it was four years. Although there was no significant difference in median duration between the two groups (p=0.113), these findings provide insights into the characteristics of the patients included in the study.

**Figure 1:**
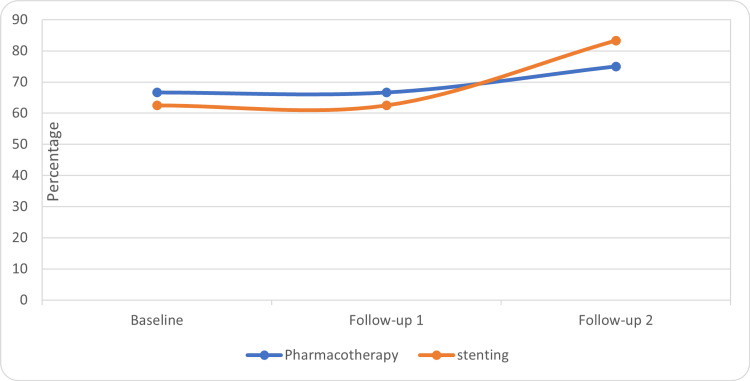
Global health scores in the treatment groups

**Figure 2:**
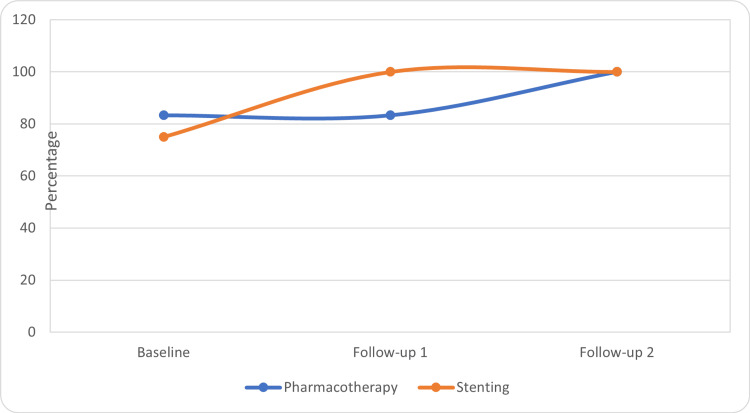
Role functioning scores in the treatment groups

**Figure 3:**
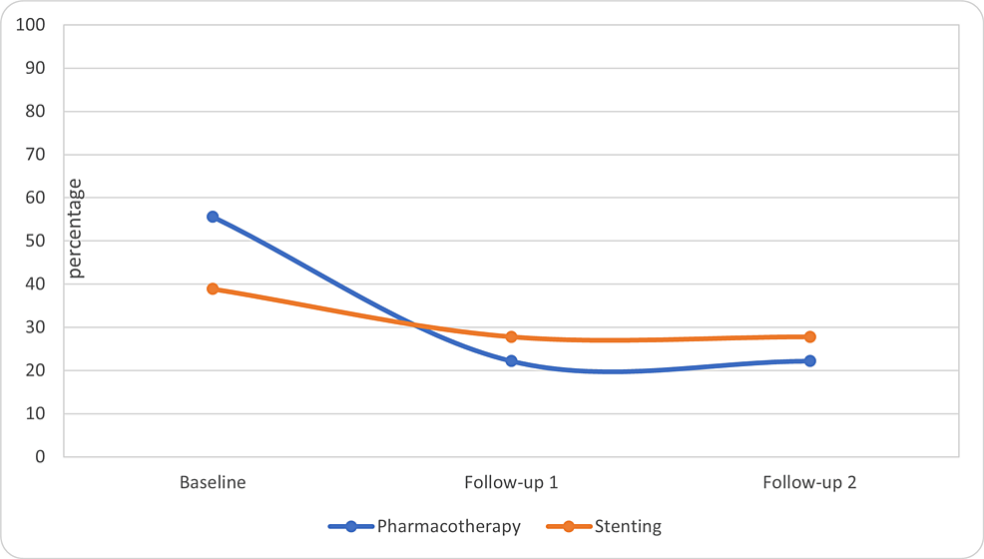
Fatigue scores in the treatment groups (median)

**Figure 4:**
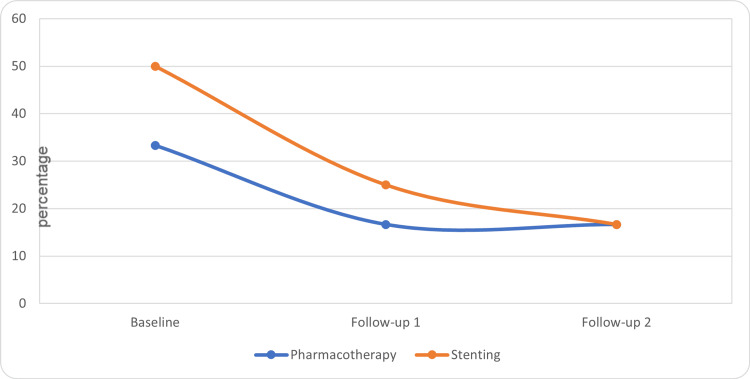
Pain scores in the treatment groups (median)

The questionnaire scales were compared within the groups for changes by using the Friedman-Wilcoxon test. In the stenting group, there was a significant improvement in the global health score at the third visit as compared to the baseline (p= 0.05) and second visit (p=0.014) (Table 1, Figure 1). There was a significant improvement in role functioning in the stenting group at the third visit as compared to the baseline (p=0.017) and at the second visit as compared to the baseline (0.027) (Table 1, Figure 2). There was no significant improvement in global health scores within the pharmacotherapy group. In the pharmacotherapy group, the fatigue score was significantly improved at the third (p=0.005) and second visit (p=0.001) as compared to the baseline (Table 1, Figure 3). There was a significant reduction in pain score at the third visit as compared to the baseline in both groups (stenting: p=0.010, pharmacotherapy: p=0.027) (Table 1, Figure 4).

**Table 1:** EORTC questionnaire scores in two treatment groups and within the group scores evaluated using the Friedman-Wilcoxon test (median (interquartile range)) *Significant difference between follow-up 2 and the baseline (p<0.05) ^¥^Significant difference between follow-up 1 and the baseline (p<0.05) ^£^Significant difference between follow-up 2 and follow-up 1 (p<0.05) EORTC: European Organization for Research and Treatment of Cancer

Scales	Groups	Baseline	Follow-up 1	Follow-up 2
Number of patients	Pharmacotherapy	18	18	17
	Stenting	12	12	12
Global health	Pharmacotherapy	58.3 (45.3-73.1)	66.6 (59.5-82.6)	75 (64.2-85.7)
	Stenting	62.5 (50.1-73.4)	62.5 (48.2-74)	83.3 (68.5-88.4)*^£^
Role functioning	Pharmacotherapy	83.3 (55.6-88.8)	83.3 (60.3-90.6)	100 (61.6-95.2)
	Stenting	75 (58.3-88.9)	100 (72.4-102.5)^¥^	100 (81-102.2)^£^
Fatigue	Pharmacotherapy	55.5 (40.2-68.3)	22.2 (13-37.9)^¥^	22.2 (15.1-41)*
	Stenting	38.8 (21.3-52.7)	27.7 (16.1-46.7)	27.7 (12-39.8)
Pain in the abdomen	Pharmacotherapy	33.3 (23.8-57.6)	16.6 (10.8-46)	16.6 (5.9-39.1)*
	Stenting	50 (22.4-52.5)	25 (17.2-57.7)	16.6 (11.9-43.6)*

Overall, the findings suggest that both treatment groups showed improvements in global health, role functioning, fatigue, and pain in the abdomen scores over the course of follow-ups. The stenting group demonstrated significant improvements in global health and role functioning compared to baseline. However, further analysis and statistical tests may be necessary to ascertain the significance of these changes.

## Discussion

There is a scarcity of studies focusing on the impact of pharmacotherapy and stenting on the quality of life of chronic pancreatitis patients. Previous research has indicated the potential benefits of pharmacotherapy, specifically enzyme replacement therapy, in chronic pancreatitis patients [18]. Furthermore, antioxidants have shown positive effects in these patients [19]. Opioids, such as morphine sulfate or fentanyl patches, have demonstrated effectiveness in reducing pain [20]. Endoscopic stent therapy has also been associated with improved clinical outcomes in chronic pancreatitis patients [21-23].

Our study provides clear evidence that both pharmacotherapy and stenting alleviate the burden of pain in chronic pancreatitis patients. Stenting, when indicated, significantly improves the global health score and role functioning. These findings were in line with the study conducted by Parhiala et al. [24].

Pharmacotherapy proves to be effective in relieving fatigue. The EORTC QLQ-C30 and QLQ-PAN26 seem suitable for evaluating chronic pancreatitis if they incorporate specific questions addressing feelings of guilt related to alcohol consumption and the challenges associated with abstaining from alcohol [25].

The role of psychological factors and psychiatric comorbidity is gaining increasing recognition in chronic pancreatitis patients. Depression, anxiety, and substance abuse disorders appear to be common, likely owing to the persistent pain and disability associated with the condition. These psychological aspects can profoundly impact the quality of life. Hence, there is a need to incorporate measurements of mood, coping abilities, and substance dependency in studies evaluating therapeutic outcomes for chronic pancreatitis [26].

In the future, newer endoscopic techniques such as extracorporeal shock wave lithotripsy and endoscopic ultrasound-guided drainage of pancreatic fluid collections may change the management algorithms and improve clinical outcomes for patients [10,27]. However, larger studies are needed to compare these modalities to standard endoscopic procedures in terms of long-term pain relief, cost-effectiveness, quality of life impact, and treatment durability [23]. Regardless of the endotherapy used, combining it with enzyme therapy and antioxidants and managing coexisting psychological comorbidities will likely deliver optimal results.

Dietary modifications and nutritional support play a pivotal role in managing chronic pancreatitis. Persistent pain often leads to reduced oral intake, while maldigestion and malabsorption contribute to micronutrient deficiencies [28]. Enteral nutrition has been found to stabilize weight loss, improve nutritional status, and reduce pain by "pancreatic rest," resulting in improved quality of life [29]. Dietary advice includes small frequent meals low in fat and high in soluble fiber. Nutritional counseling by a specialist dietician is recommended for all patients [30].

An emerging area that requires further research is the impact of chronic pancreatitis on employment, productivity, and socioeconomic status. Since this condition often affects young individuals in their peak productive years, it likely has a substantial indirect economic burden. One research indicated that more than 70% of individuals suffering from chronic pancreatitis experienced an impact on their professional lives, and a significant proportion has reported lower household incomes for these patients [31]. Assessing measures such as work impairment, job loss risk, and disability claims will provide greater insights into the wider impairments caused by chronic pancreatitis that potentially strain quality of life.

This study has several notable strengths. Firstly, it utilizes the validated EORTC QLQ-C30 questionnaire, which is a reliable tool for health-related quality of life assessment in clinical trials. Secondly, the study design prospectively compares two standardized interventions: pharmacotherapy alone and stenting with pharmacotherapy. The follow-up at regular intervals over six months also allows for a longitudinal analysis. Thirdly, the eligibility criteria were clearly defined, with the exclusion of cancer patients and those with cognitive issues or comorbidities, improving internal validity. Fourthly, the modest sample size still enabled statistically significant differences between groups to emerge, proving that the study was adequately powered. Fifthly, patient-centered outcomes such as quality of life were evaluated rather than only clinical or biochemical markers. Lastly, advanced nonparametric statistical tests were appropriately used for analysis. These methodological strengths lend credibility to the study findings demonstrating quality of life improvements across domains with both interventions.

Our study has several limitations that need to be acknowledged. Firstly, the sample of chronic pancreatitis patients included in the study was obtained from a large tertiary care referral center, which may introduce selection bias and limit the generalizability of the findings to patients seen in primary care or community gastroenterology practices. Therefore, the results may primarily reflect individuals with more severe diseases. Secondly, the study had a relatively small sample size and a short duration of observation. This may limit the statistical power and the ability to capture long-term effects or changes over time accurately. Thirdly, there are other factors that can influence health-related quality of life, such as economic status and environmental factors. These attributes vary among patients and may not have been fully adjusted in our statistical analysis.

Despite these limitations, our study contributes valuable insights into the impact of chronic pancreatitis on functional status and overall well-being. The data highlights that in addition to the cardinal symptom of pain, less frequently mentioned symptoms such as fatigue, loss of appetite, and financial difficulties have a significant impact on health-related quality of life. Standardized assessments of health-related quality of life have the potential to enhance physicians' understanding of their patients' challenges and needs [32]. The findings from this study can serve as a foundation for the development of disease-specific questionnaires needed for longitudinal studies involving patients with chronic pancreatitis [33].

## Conclusions

In summary, the available studies indicate positive outcomes for medication and stenting in individuals with chronic pancreatitis. Antioxidants, enzyme replacement therapy, opioids, and endoscopic stent therapy have all shown potential benefits in improving the quality of life of these patients. Our own study supports these findings, demonstrating that both medication and stenting effectively reduce pain and improve aspects of quality of life, such as role functioning and global health scores. However, it is important to recognize the broader impact of chronic pancreatitis on patients' well-being and functional status. Symptoms beyond pain, such as fatigue, loss of appetite, and financial difficulties, significantly affect health-related quality of life. Standardized assessments of quality of life can enhance physicians' understanding of these challenges and guide future research using disease-specific questionnaires. Further research is needed to fully comprehend the mechanisms through which medication and stenting contribute to the improved quality of life of individuals with chronic pancreatitis. Nonetheless, the existing evidence suggests that these interventions hold promise in enhancing the well-being and functional outcomes of patients with this condition.
